# Prevalence of pathogenetic MC4R mutations in Italian children with early Onset obesity, tall stature and familial history of obesity

**DOI:** 10.1186/1471-2350-10-25

**Published:** 2009-03-12

**Authors:** Nicola Santoro, Grazia Cirillo, Zhimin Xiang, Rita Tanas, Nella Greggio, Giuseppe Morino, Lorenzo Iughetti, Alessandra Vottero, Alessandro Salvatoni, Mario Di Pietro, Antonio Balsamo, Antonino Crinò, Anna Grandone, Carrie Haskell-Luevano, Laura Perrone, Emanuele Miraglia del Giudice

**Affiliations:** 1 Dipartimento di Pediatria "F. Fede", Seconda Università degli Studi di Napoli, Napoli, Italy; 2 Department of Medicinal Chemistry, University of Florida, Gainesville, FL 32610, USA; 3 Azienda Ospedaliero-Universitaria Arcispedale S. Anna, Ferrara, Italy; 4 Dipartimento di Pediatria – Università di Padova, Padova, Italy; 5 Ospedale Pediatrico Bambin Gesù, Roma, Servizio di dietologia clinica, Roma, Italy; 6 Università di Modena e Reggio Emilia, Dipartimento di Pediatria, Modena, Italy; 7 Università di Parma, Clinica Pediatrica, Dipartimento dell'età evolutiva, Parma, Italy; 8 Università dell'Insubria, Clinica Pediatrica, Dipartimento di Scienze Cliniche e Biologiche, Italy; 9 Ospedale S. Liberatore di Atri, UO di Pediatria, Teramo, Italy; 10 Policlinico S. Orsola-Malpighi, Dipartimento di Scienze pediatriche, Bologna, Italy; 11 Ospedale Pediatrico Bambin Gesù, Roma, Paediatric and Autoimmune Endocrine Diseases Unit, Roma, Italy

## Abstract

**Background:**

Melanocortin-4-receptor (MC4R) mutations represent the most frequent genetic cause of non-syndromic early onset obesity. Children carrying MC4R mutations seem to show a particular phenotype characterized by early onset, severe obesity and high stature. To verify whether *MC4R *mutations are associated with this particular phenotype in the Italian pediatric population, we decided to screen the MC4R gene in a group of obese children selected on the basis of their phenotype.

**Methods:**

To perform this study, a multicentric approach was designed. Particularly, to be enrolled in the study subjects needed to meet the following criteria: Body mass index ≥ 3 deviation scores according to age and sex, familiar history of obesity (at least one parent obese), obesity onset before the 10 years old, height ≥ 2 deviation scores. The coding region of MC4R gene was screened in 240 obese children (mean age 8.3 ± 3.1, mean BMI 30.8 ± 5.4) and in 200 controls (mean age 8.1 ± 2.8; mean BMI 14.2 ± 2.5).

**Results:**

Three mutations have been found in five obese children. The S127L (C380T), found in three unrelated children, had been described and functionally characterized previously. The Q307X (C919T) and the Y332H (T994C) mutations were found in two patients. Functional studies showed that only Q307X impaired protein function.

**Conclusion:**

The low prevalence of *MC4R *mutations (1.6%) in this group of obese children selected according to the obesity degree, the tall stature and the family history of obesity was similar to the prevalence observed in previous screenings performed in obese adults and in not phenotypically selected obese children.

## Background

Prevalence of obesity has dramatically increased in children and adolescents in the past 25 years [1] and studies concerning molecular basis of obesity have been encouraged.

Melanocortinergic system represents the most interesting known system involved in the central regulation of body weight. Blockade of the melanocortin signalling pathway leads to hyperphagia, reduced energy expenditure and, ultimately, obesity [2]. Proopiomelanocortin (POMC) represents a key step in the anorexigenic signaling cascade of leptin [3]. In the hypothalamus, the POMC derived peptides α-MSH and β-MSH bind the melanocortin-4-receptor (MC4R), a seven transmembrane receptor [4,5] causing a reduction of appetite and an increase of energy expenditure. Regulation of energy homoeostasis through this pathway is highly susceptible to quantitative variations in *MC4-R *expression or function. These variations may be consequence of a reduced ligand binding or of a reduced receptor expression. Molecular screenings allowed to identify mutations on *POMC *and *MC4R *associated with early onset obesity [6-8].

*MC4R *mutations represent the most frequent cause of non syndromic early onset obesity, with prevalence ranging from 0.5% to 5% [9,10]. Several mutations on *MC4R *have been found and functionally characterized in humans. Clinical characteristics of subjects carrying mutations on *MC4R *have been carefully described [10]. Their particular features are early onset, severe, obesity, accelerated height velocity (height is usually more than 2 DS), advanced bone age and hyperinsulinemia.

Surprisingly, recent studies on large cohorts of obese adults and children described mutations in the obese patients, but also in the normal weight individuals [11-13], and failed to establish that the early onset of obesity [12] as well as the tall stature [13] of the obese children are specific clinical characteristics of functionally relevant heterozygous *MC4R *mutation carriers.

In a previous study we observed a prevalence of *MC4R *mutations among Italian obese children and adolescent of 0.5%, lower than that previously showed by other authors in obese children from different geographic areas [9]. The low prevalence that we found was attributed to the selection criteria and to the method used for molecular screening [14]. To understand whether the previous data were biased by not strict selection criteria in patients recruitment, we have screened MC4R in a group of Italian obese children selected, with a multicentric approach, on the basis of their phenotype (i.e.; familiar history of obesity, BMI ≥ 3 deviation scores, obesity onset before the 10 years old, height ≥ 2 deviation scores).

## Methods

### Recruitment criteria

The Childhood Obesity Study Group of the Italian Society of Pediatric Endocrinology and Diabetes (SIEDP) was involved in the study and the following nine childhood obesity services from nine different cities participated: Ferrara, Varese, Bologna, Modena, Parma and Padova (Northern Italy), Roma and Atri, (Central Italy) and Napoli (Southern Italy).

Recruitment started on January 2005 and stopped on January 2006. To be enrolled in the study children needed to meet the following criteria: a BMI ≥ 3 SD (severe obesity) according to age and sex, familiar history of obesity (at least one parent should be obese or ex obese), obesity onset before the 10 years old, height ≥ 2 deviation scores. All patients data and blood samples were sent to the Department of Pediatrics of the Second University of Naples. The ethical committee of the Second University of Study of Naples approved the study. Informed consent was obtained by parents.

### Clinical data

Body weight was measured by a balance beam scale, the subject being undressed, height was measured by a Harpenden Stadiometer and BMI was calculated. Standard deviations scores for BMI was calculated by using the LMS method [15]. The LMS method fits growth standards to all forms of anthropometry by making the simple assumption of a skew normal distribution. Standard deviation of height (SD-height) and pubertal stage were evaluated according to Tanner [16]. Waist circumference was measured with an anelasticated tape, the subject being in standing position; the tape is applied horizontally midway between the lowest rib margin and the iliac crest. To assess the age of obesity onset during early childhood, the records of the patients were reviewed. In these records the anthropometric measurements made in the pediatrician's surgeries within the ambit of children health balances made annually are reported [17].

### The control group

A control group composed by 200 age and sex matched lean children was recruited as previously shown [18]. Briefly, lean children who were age and sex matched and belonged to the region of Napoli were recruited as controls (mean age, 10.7 ± 2.2 yr; mean BMI z-score, 0.5 ± 0.4). They consulted the Department of Pediatrics of the Second University of Naples for presumed diseases and were found to be normal. Informed consent from parents was obtained before entry in the study.

### Genotyping

#### Melanocortin- 4-receptor (*MC4R*)

Genomic DNA was collected from nucleated white blood cells.

Amplification of the *MC4R *coding region was performed using five primer pairs and the condition previously described [9]. PCR products were analysed by an automatic sequencer (ABI PRISM 310, Perkin Elmer, USA).

### Functional Study

#### Materials

Peptides used in this study, *α*-MSH, 4-norleucine-7-**D**-phenylalanine (NDP)-MSH, ACTH(1–24), *β*-MSH, *γ*_2_-MSH, were purchased from commercial sources (Bachem, Terrance, CA, USA). The melanocortin tetrapeptide JRH887–9 (Ac-His-**D**-Phe-Arg-Trp-NH_2_) was synthesized as previously reported [19].

#### hMC4R *in vitro *receptor mutagenesis

The human wild-type (WT) N-terminal Flag-tagged hMC4R cDNA mutagenesis was performed as described previously [19]. Amino acid modifications of the hMC4R were introduced using a complementary set of primers containing the nucleotide mutation(s) resulting in the desired residue change. The construction of hMC4R containing the desired mutant has been described previously [19]. Complete Flag-hMC4R sequences were confirmed free of PCR nucleotide base errors by DNA sequencing (University of Florida sequencing core facilities).

#### cAMP-based functional bioassay

Human embryonic kidney-293 cells stably expressing WT and mutant receptors were transfected with cAMP response element (CRE)/*β*-galactosidase reporter gene as previously described [19]. Briefly, forty-eight hours post-transfection, the cells were stimulated with the peptide for *α*-MSH, NDP-MSH, and ACTH(1–24) and the peptide for *γ*_2_-MSH, *β*-MSH, and JRH887–9 or forskolin control in assay medium for 6 h. Subsequently, substrate buffer [60 mM sodium phosphate, 1 mM MgCl_2_, 10 mM KCl, 5 mM *β*-mercaptoethanol, 2 mg/mL of *o*-nitrophenyl-*β*-D-galactopyranoside (ONPG)] was added to the cell lysate plates, and were incubated at 37°C. The sample absorbance was measured and data points were normalized both to the relative protein content and non-receptor-dependent forskolin values. Assays were performed using duplicate data points and repeated in at least four independent experiments. Means and standard errors (SE) are reported.

#### Receptor-binding studies

Human embryonic kidney-293 cells stably expressing the WT and mutant receptors were maintained as described above. The peptide NDP-MSH was used to competitively displace the ^125^I-radiolabeled peptides NDP-MSH. Dose-response curves (10^-6 ^to 10^-12 ^M) and IC_50 _values were generated and analyzed by non-linear least-squares analysis [19]. The percentage total specific binding was determined based upon the non-specific values obtained using 10^-6 ^M NDP-MSH for the radiolabeled peptide. Each experiment was performed using duplicate data points and repeated in at least two independent experiments.

#### FACS analysis of wild-type and mutant Flag-tagged hMC4 receptors

Fluorescence-activated cell sorting (FACS) analysis of N-terminally Flag-tagged WT and mutant hMC4R was performed as described previously [19]. For cell surface and intracellular detection of the Flag-hMC4Rs an allophycocyanin (APC)-conjugated anti-Flag monoclonal antibody (Prozyme, San Leandro, CA, USA) was used. To detect the total (surface and intracellular) receptor expression cells were subsequently permeabilized with saponin buffer and stained with the APC-conjugated anti-Flag monoclonal antibody. Unlabeled cells were used to set the background fluorescence staining for these analyses. BD Biosciences (San Jose, CA, USA) FACS Calibur flow cytometers were used to collect both stained cell percentages (surface and total) and mean fluorescence data were measured from a minimum of 10 000 collected events per sample. Experiments were repeated three independent times and the mean is reported.

## Results

We enrolled 240 obese children and adolescents (135 girls) with a mean age 8.3 ± 3.1 years, a mean age at onset of obesity of 2.8 ± 2.0 years, the mean SD-height was 2.3 ± 0.2, mean BMI 30.8 ± 5.4 and z-score BMI 4.2 ± 0.9.

**Figure 1:**
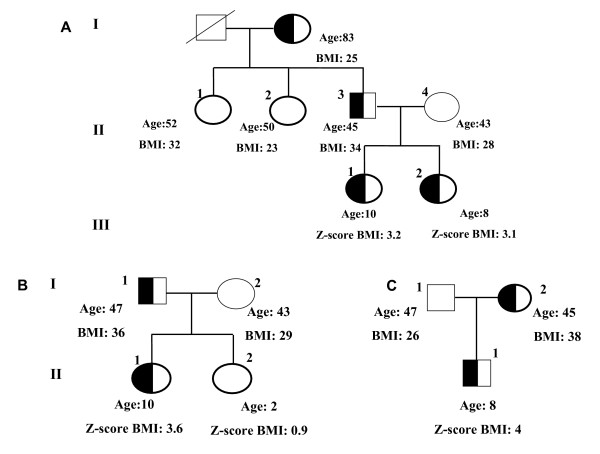
**Family trees of the probands carrying the S127L variant**. This variant was found in three unrelated subjects from three different obesity Services. **A **show the family tree of subject 1 (see Table 1). The figure shows that all the carriers but the subject I 2 (grandmother) were obese. **B **and **C **show the family trees of patients 2 and 3, respectively (see Table 1). The half black square indicates males heterozygotes for the mutation, the half black circle indicates the females heterozygotes for the mutation.

**Figure 2:**
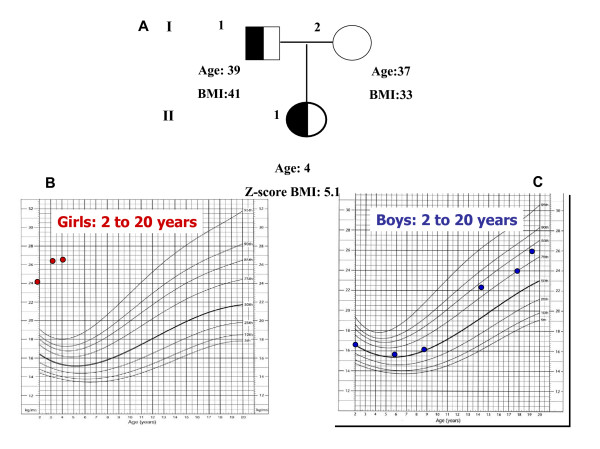
**A. The essential family tree (father, mother and daughter) of the proband carrying the Q307X mutation is shown**. **B **and **C **show the BMI chart for the subject II 1 (proband) and for the subject I 1 (the father), respectively. Both carry the mutation, but, while subject II 1 shows an early onset of obesity, the subject I 1 became obese after he was 20 years old. The half black square indicates males heterozygotes for the mutation, the half black circle indicates the females heterozygotes for the mutation.

Molecular screening of the *MC4R *showed the substitution of a serine with a leucine at the codon 127 (S127L) consequent to the substitution of a cytosine in position 380 with a thymidine, a substitution of the glutamine with a stop codon at the codon 307 (Q307X) caused by the substitution of a cytosine with a thymidine in position 919 (C919T) and the substitution of a tyrosine with a histidine at the codon 332 (Y332H) caused by the substitution of a thymidine with a cytosine in position 994 (T994C). The S127L mutation was found in three non-consanguineous individuals from three different centers (Modena, Bologna, Napoli), the two mutations, Y332H and Q307X were found in a child from Napoli and in a child from Ferrara, respectively (table 1). Four out of five families were investigated and mutations co-segregated with obesity in 9 out of 10 individuals harboring a variant (figures 1 and 2).

**Table 1:** Clinical features of obese children with MC4R mutations

**Mutation (sex)**	**S127L (M)**	**S127L (F)**	**S127L (F)**	**Q307X (F)**	**Y332H (F)**
**Age (years)**	8	10	10	3	11
**Age at obesity onset (years)**	2	4	7	1	3
**z-score BMI**	3.2	3.6	4.0	5.1	4.0
**Height-sds**	2.0	2.1	2.4	2.0	2.2

None lean subject showed the above described mutations. Four obese subjects (3 from Napoli and one from Modena) and three lean controls (males) showed a common, polymorphism (I103V) previously described as negatively associated with obesity [10].

**Figure 3:**
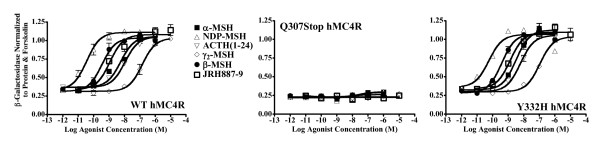
**Functional Agonist Data**. Illustration of the activity of α-MSH, ACTH_1–24_, β-MSH, γ2-MSH, and synthetic agonist JRH887–9 at the wild type MC4R, Q307X MC4R and Y332H MC4R. Agonists did not evocate any response by binding the Q307X MC4R. When the agonists were binding the Y332H MC4R the evocated response was similar to that observed for the wild type.

When the ability of the Q307X and Y332H mutated MC4Rs to generate cAMP in response to increasing concentrations of α-MSH was tested, the Q307X *MC4R *did not evocate any response, while the response evocated by the agonist binding the Y332H *MC4R *(6.29 ± 1.93) was similar to that evocated by binding the wild type receptor (6.85 ± 3.23) (figure 3)

To evaluate if some ligand may pharmacologically rescue the agonist response of the mutated Q307X MC4R, the endogenous melanocortin agonists ACTH_1–24_, β-MSH, γ2-MSH, and the synthetic agonist JRH887–9 were tested. None was able to rescue the functional activity of the mutated protein (figure 3).

**Figure 4:**
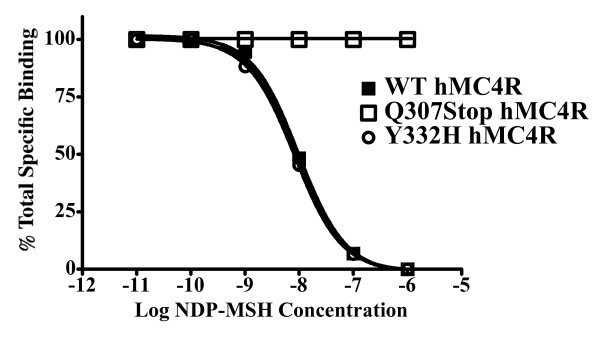
**Binding Data**. Competitive displacement binding affinity studies of the polymorphic hMC4Rs examined in this study. I^125^NDP-MSH was used to competitively displace non-labeled NDP-MSH in a dose-response fashion. The endogenous agonists were unable to stimulate the Q307X MC4R at up to 1 μM concentrations.

Binding data obtained using the radiolabelled NDP-MSH showed that, in the case of the Q307X mutation, the endogenous agonist was unable to stimulate the receptor (figure 4).

**Figure 5:**
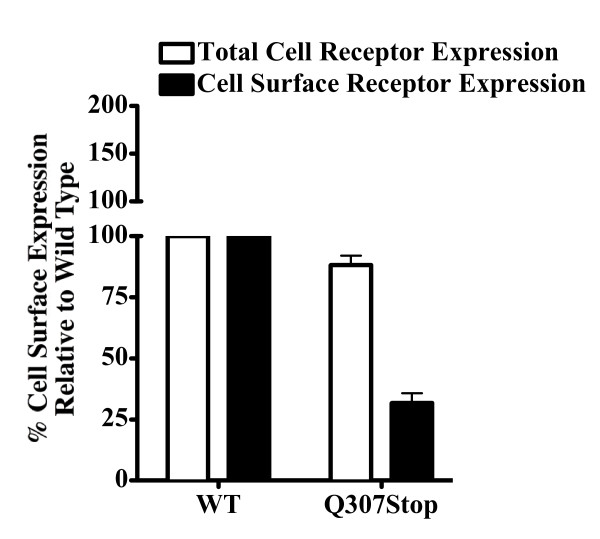
**FACS Data**. Fluorescence activated cell sorting analysis (FACS) of the MC4R Q307X mutation expressed in HEK-293. The total cell receptor expression levels were determined using permeabilized cells measuring both cell surface and intracellular protein expression. The cell surface expression levels were determined using non-permeabilized cells. Cell expression levels are presented relative to the wild type human-MC4R control. Total Q307X expression was 88%, surface expression was 31% and intracellular retention was about 57%.

As intracellular retention of mutated MC4Rs is a common obesity-causing defect, to allow for the rapid evaluation of cell surface expression of Q307X relative to total expression of the receptor in individual transiently transfected cells, a method based on immunostaining and fluorescence detection by flow cytometry has been used. As compared to the wild type expression, total Q307X expression was 88%, surface expression was 31% and, therefore, intracellular retention was about 57% (figure 5).

Given that the Y332H variant did not affect the function of MC4R, this latter mutation was not included in the calculation of the prevalence of pathogenetic relevant MC4R gene mutations in our population, that can be consequently considered 1.6% (4 out of 240 patients).

## Discussion

### *MC4R *mutations functional analysis

The S127L variant has been functionally studied previously [20], while the Q307X has been described but not functionally characterized [21]. The S127L impairs the signaling in response to α-, β- and γ-MSH [20], but not the MC4R expression on cell surface [14] and is, therefore, highly likely to be causative, rather than incidental.

Of the two mutations, Q307X and Y332H, only the Q307X showed a functional relevance. At our best knowledge, at date, this is the most C-terminal nonsense mutation on *MC4R*. This mutation produces a protein lacking the last 26 amino acids. Among these, five are residues at 100% conserved during evolution in vertebrates [22]. The C-terminal tail has previously been identified as a region important for targeting to the plasma membrane. Accordingly, we have demonstrated some intracellular retention for the Q307X protein. This finding is consistent with those of Ho and MacKenzie [23] who demonstrated that an artificial receptor lacking amino acids 306–319 within the C-terminal tail was unable to traffic to the plasma membrane [23]. Rather surprisingly given its location in the C-terminal tail of the receptor, the Q307X mutant showed also a decreased binding affinity for agonist. This unexpected finding has been yet showed in another *MC4R *mutation affecting the tail of the receptor (I316S) [24]. It is conceivable that in both cases the mutations may induce a sufficient alteration of the receptor tertiary structure of the ectodomain, or that they may affect interaction with intracellular kinases or other enzymes which might post-translationally modify the receptor and modulate receptor-ligand interaction [24]. Concluding, the impaired signaling of the Q307X mutation is due to a combination of defects, i.e.; both reduced cell surface expression and decreased affinity of the receptor for *α*-MSH.

### *MC4R *mutations prevalence analysis

Mutations on *MC4R *gene represent the most frequent cause of monogenic non-syndromic obesity, with prevalence, among obese children, reaching in some cases the 5% [10]. In a previous report we observed a prevalence of pathogenetic *MC4R *gene mutations in a population of Italian obese children and adolescents of about 0.5%. Selection criteria, particularly the lower mean BMI of the population investigated compared to the BMI of the other groups of patients studied, have been claimed to explain this finding [24].

The present is the first study which aims to find mutated children specifically selecting them on the basis of the particular phenotypic characteristics usually recognized in the patients carrying *MC4R *variations (i.e.; at least one parent obese or ex obese, obesity onset before 10 years of age, BMI more than 3 DS and height more than 2 DS) [11]. Anyway, we have to recognize that studies trying to find a phenotype-genotype correlation were not conclusive. In fact, studies showing mutations in the obese as well as in the normal weight individuals [11-13] failed to establish that the early onset of obesity [12] and the tall stature [13] of the obese children are specific clinical characteristics of functionally relevant *MC4R *mutations. On the other hand, studies confuting the association between the *MC4R *mutations and early onset obesity describe populations of obese adults without objective data on whether the obese adults had been obese as children or not [12,13]. Prevalence of MC4R mutations in this cohort of phenotypically selected children (1.6%) appears similar to the 1.8% reported in a previous paper investigating a large cohort, not phenotypically selected, of European obese children [13].

Prevalence of MC4R mutations observed in the present study is also similar to that showed in Italian obese adults, where it ranges from 1.8% [25] to 2.5% [26]. This observation is consistent with a recent report of Lubrano-Bethelier et al. showing that obese adults carriers of functionally relevant *MC4R *mutations do not specifically present with a history of early onset obesity [12] and with the report of Stutzman et al showing a prevalence of MC4R mutations of 1.6% among obese adults [13].

Reduced penetrance and variable expressivity of obesity has been found to be associated with MC4R mutations. In fact, whereas some found a 100% penetrance of early onset obesity in heterozygous proband, others have described obligate carriers who were not obese also among children [13]. The variable penetrance and expressivity of obesity in heterozygous individuals argues that the MC4R acts in concert with a number of other genes to regulate energy storage under presumed conditions of a sedentary lifestyle and high-fat diet [27].

It is important to note that pathogenic *MC4R *mutations are "private", with individual mutations having a very low frequency in any population [28]. We observed the same mutation in three different non-consanguineous subjects from three different parts of Italy. Moreover this mutation has not been observed in the three previous screening on Italian population [9,24,25]. This observation is not surprisingly considering that the S127L variant has been described by other groups [10,24].

We did not compare our cohort of specifically selected obese children with a group of equivalently obese children without increased stature. This limitation does not allow us to firmly conclude that there are no phenotypic characteristics of MC4R mutations other than obesity *per se*.

### *MC4R *V103I polymorphism

The previously described polymorphism A307G resulting in the substitution of Val with Ile at codon 103 within the MC4R TM2 was detected at the heterozygous state in 4 obese subjects (1.6%) and in 3 lean controls (1.5%). Previous studies provided the evidence for a negative association of the I103 allele with obesity [29-31]. Herein we did not observe a statistically significant difference in the prevalence of this variant between lean and obese subjects, but as noted by Geller et al. none of the individual studies included in previous meta-analysis rendered a p value < .05 [30]. Thus, although this observation cannot be considered conclusive, our observation may be used for further meta-analysis.

## Conclusion

In conclusion, we have reported the first MC4R molecular screening performed in a population of obese children stringently selected on the basis of their phenotype. The prevalence of *MC4R *mutations (1.6%) in this group of obese children selected according to the obesity degree, the tall stature and the family history of obesity was similar to the prevalence observed in previous screenings performed in obese adults and in not phenotypically selected obese children.

## Competing interests

The authors declare that they have no competing interests.

## Authors' contributions

NS analytic framework and writing the manuscript, EmdG had the primary responsibility for protocol development and writing the manuscript. GC carried out the molecular screening. AG participated in the development of the protocol, patients' recruitment and in the preliminary data analysis. CH-L carried out the laboratory work concerning the functional analysis. ZX was responsible for the laboratory work concerning the functional analysis. LP supervised the design and execution of the study. RT, NG, GM, LI, AV, AS, MDP, AB and AC were responsible for selecting and evaluating the patients in the respective centers. All authors read and approved the manuscript.

## Pre-publication history

The pre-publication history for this paper can be accessed here:


